# Prognostic factors of patients with extremity myxoid liposarcomas after surgery

**DOI:** 10.1186/s13018-019-1120-2

**Published:** 2019-03-28

**Authors:** Jiaqi Wu, Shengjun Qian, Libin Jin

**Affiliations:** Department of Orthopaedics, Centre for Orthopaedic Research, Orthopedics Research Institute of Zhejiang University, The Second Affiliated Hospital, Zhejiang University School of Medicine, 88 Jiefang Road, Hangzhou, 310000 Zhejiang People’s Republic of China

**Keywords:** Myxoid liposarcoma, Extremity, Prognostic factors, Treatment

## Abstract

**Background:**

Extremity myxoid liposarcoma (MLS) is a rare soft tissue sarcoma in adults. We performed this study to define distinctive clinical features of extremity MLS by assessing prognostic factors.

**Methods:**

Between 1973 and 2015, 1756 patients with extremity MLS who underwent surgical resection were retrieved from the Surveillance, Epidemiology, and End Results (SEER) database of the US National Cancer Institute. Both overall survival (OS) and cancer-specific survival (CSS) were assessed using the Kaplan–Meier method (to obtain OS and CSS curves) and a Cox proportional hazards regression model.

**Results:**

Of the 1756 patients with extremity MLS, the mean and median patient age at diagnosis were 47 and 45 years, respectively. More than half (*n* = 1027, 58.5%) of the patients were male. In terms of location, 10.5% tumors were located in the upper limbs and 89.5% in lower limbs. All patients received local surgery, and about half of the patients (57.2%) received radiation treatment. The 5- and 10-year OS rates of the entire cohort were 86.4% and 75.9%, respectively. The 5- and 10-year CSS rates were 90.5% and 85.2%, respectively. On multivariate analysis, older age, male gender, high tumor grade, and tumor size > 10 cm were found to be independent risk factors of both decreased OS and CSS. Year of diagnosis ≥ year 2000 was significantly associated with an increased CSS. In addition, radiation treatment failed to become an independent risk factor for either OS or CSS.

**Conclusion:**

We identified age, gender, tumor grade, year of diagnosis, and tumor size as independent prognostic factors for OS and CSS in patients with extremity MLS.

## Background

Liposarcoma (LPS) is the most common soft tissue sarcoma (STS) of the extremities in adults, and it is classified into four subtypes according to the 2013 WHO classification, namely, dedifferentiated, myxoid, pleomorphic, and not otherwise specified [1]. The round cell LPS type has been included in the myxoid LPS. Myxoid liposarcoma (MLS) is the second most common type of liposarcoma, accounting for 15–20% of all liposarcomas [2]. The demographic data and clinical outcomes of extremity LPS or STS are well documented [3–7]. Surgical resection is the mainstream treatment for extremity LPS. However, adjunct radiotherapy (RT) plays an important role in the treatment of extremity LPS. Combination of surgery and adjuvant RT has been increasingly used to improve local control of the disease and decrease the recurrence [8]. In particular, MLS has been reported to be relatively radiosensitive compared with other subtypes [8]. To our knowledge, there are currently no studies exploring the effect of RT on improving the survival of patients with extremity MLS. Extremity LPS is not a single entity, and the prognosis varies according to the pathological type [9]. Predicting the prognosis for patients with extremity MLS can facilitate appropriate treatment decisions. In addition, no large-scale study exploring the prognostic factors of extremity MLS patients has been reported.

This study was performed to investigate the prognosis of this special cohort and determine the independent prognostic factors, based on the Surveillance, Epidemiology, and End Results (SEER) program database of the National Cancer Institute.

## Materials and methods

### Patient population

This study followed standard guidelines and was approved by the Ethics Committee of the Second Affiliated Hospital, Zhejiang University School of Medicine. From 1973 to 2015, a total of 1990 patients diagnosed with extremity MLS were identified from the SEER program database, using the case-listing session procedure. The database is publicly available and does not include unique patient identifiers.

**Fig. 1:**
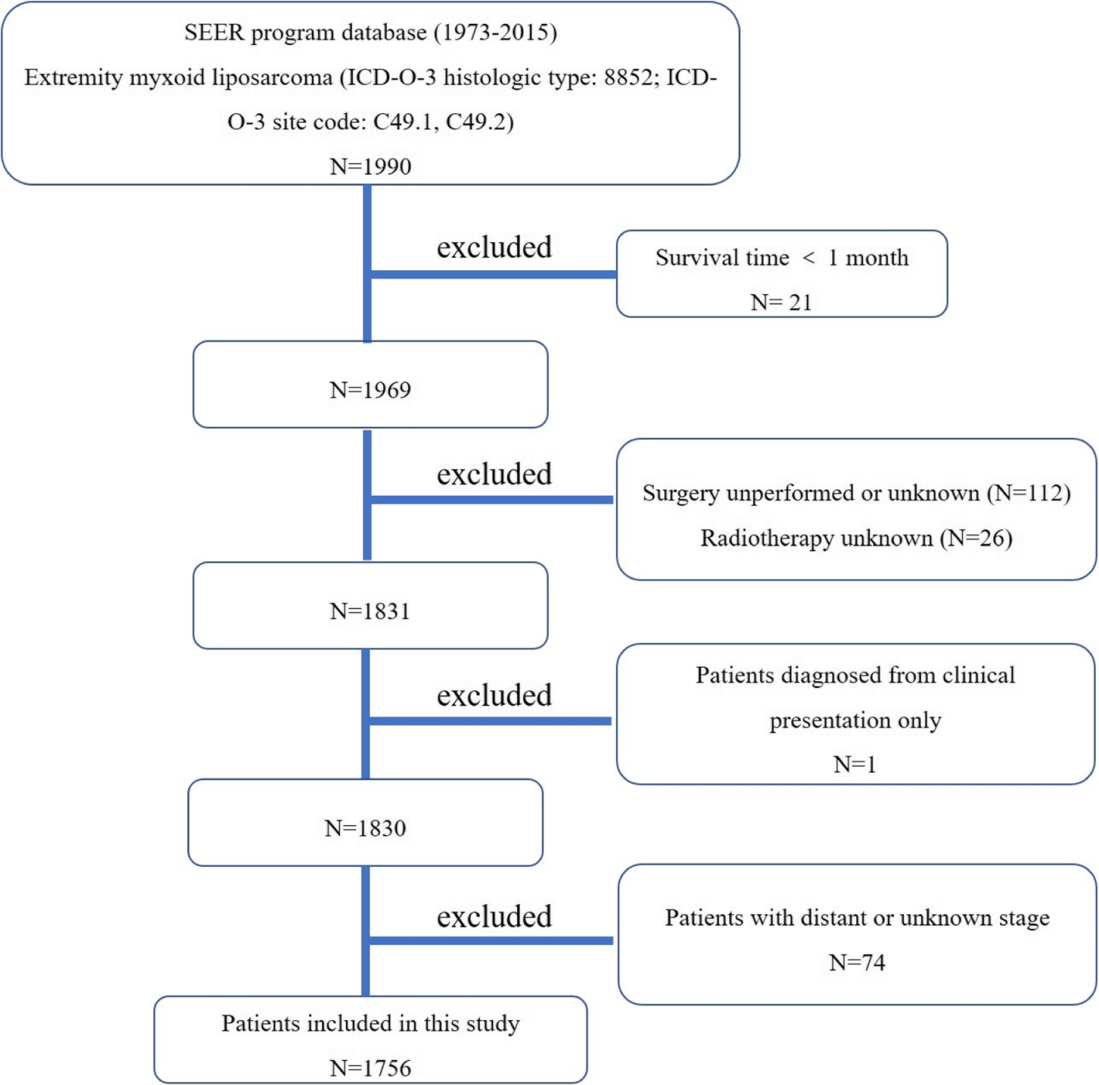
The flow chart for selection of study population. (Abbreviations: SEER, Surveillance, Epidemiology, and End Results; ICD-O-3, international classification of diseases for oncology, 3rd edition)

First, the International Classification of Diseases for Oncology, 3rd edition (ICD-O-3) was used to identify patients with extremity MLS (ICD-O-3 histologic type: 8852; ICD-O-3 site code: C49.1 and C49.2). Patients with survival time < 1 month were excluded. One hundred twelve patients with no or unknown surgery and 26 with unknown radiotherapy were excluded. All patient diagnoses were confirmed histologically, based either on biopsy results or the surgical specimen. One patient diagnosed only on the basis of the clinical presentation was excluded. Seventy-four patients with distant or unknown stage were also excluded. The inclusion criteria are shown in Fig. 1. Data extracted from the SEER database included age, gender, tumor site, tumor grade, tumor stage, tumor size, surgical treatment, radiation treatment, cause of death, and survival time. Surgery or radiation treatment for tumors in this study refers to treatment for local primary tumors. This study did not include the patients with round cell LPS because the round cell component is an effective prognostic factor [10, 11].

### Statistical methods

The SPSS statistical software (version 22.0) and Microsoft Excel 2016 were used to analyze the data. Overall survival (OS) was defined as the time from diagnosis to death from any cause, and cancer-specific survival (CSS) was defined as the time from diagnosis to death specific to the cancer-related diagnosis. Univariate analyses were performed using the Kaplan–Meier method with the log-rank test. Survival curves were generated by the Kaplan–Meier method and compared by log-rank test. Observations were censored if the patient was alive at the time of the last follow-up. Multivariate analysis was performed to determine the independent predictors of OS and CSS with Cox proportional-hazard regression analyses. The hazard ratios (HRs) and corresponding 95% confidence intervals (CIs) were calculated to show the effect of factors on OS and CSS. Differences were deemed statistically significant if *p* < 0.05.

## Results

### Demographic and clinical characteristics of patients with extremity MLS

From 1973 to 2015, data for a total of 1756 patients with extremity MLS who met the inclusion criteria were collected from the SEER database. Demographic and clinical characteristics of patients are listed in Table 1. The mean and median patient age at diagnosis were 47 ± 17 years and 45 years, respectively (ranging from 1 to 101). More than half (*n* = 1027, 58.5%) of the patients were male. More than two thirds of the cases were diagnosed after year 2000. In terms of location, 10.5% of the tumors were located in the upper limbs, and 89.5% in the lower limbs. Nine hundred eighty-eight (56.3%) of the tumors were categorized as low grade. Information on the tumor size was available in 87% of the cases and was categorized into four groups. In addition, all patients received local surgery, and about half of the patients (57.2%) received radiation treatment. Ultimately, 463 patients (26.4%) died, of whom 195 died of cancer. The 5- and 10-year OS rates of the entire cohort were 86.4% and 75.9%, respectively. The 5- and 10-year CSS rates were 90.5% and 85.2%, respectively (Table 1).

**Table 1:** Demographic and clinical characteristics of 1756 patients with myxoid liposarcoma of extremities after surgery identified in the SEER database from 1973 to 2015

Category	Value
Mean ± SD age (years)	47 ± 17
Median age (years)	45
Age (years)	
< 30	253 (14.4%)
30–60	1099 (62.6%)
> 60	404 (23.0%)
Gender	
Female	729 (41.5%)
Male	1027 (58.5%)
Year of diagnosis	
< 2000	527 (30.0%)
≥ 2000	1229 (70.0%)
Location	
Upper limb	185 (10.5%)
Lower limb	1571 (89.5%)
Tumor size	
Mean (cm)	11
Median (cm)	10
< 5 cm	232 (13.2%)
5–10 cm	609 (34.7%)
> 10 cm	686 (39.1%)
Unknown	229 (13.0%)
Tumor grade^a^	
Low	988 (56.3%)
High	232 (13.2%)
Unknown	536 (30.5%)
Radiation treatment	
Yes	1004 (57.2%)
No	752 (42.8%)
Dead	
Yes	463 (26.4%)
No	1293 (73.6%)
5-year OS rate	86.4%
5-year CSS rate	90.5%
10-year OS rate	75.9%
10-year CSS rate	85.2%

*SD* standard deviation, *OS* overall survival, *CSS* cancer-specific survival

^a^Low: grade I (well differentiated) and grade II (moderately differentiated). High: grade III (poorly differentiated) and grade IV (undifferentiated anaplastic)

### Univariate analyses of variables associated with OS or CSS among patients with extremity MLS

**Fig. 2:**
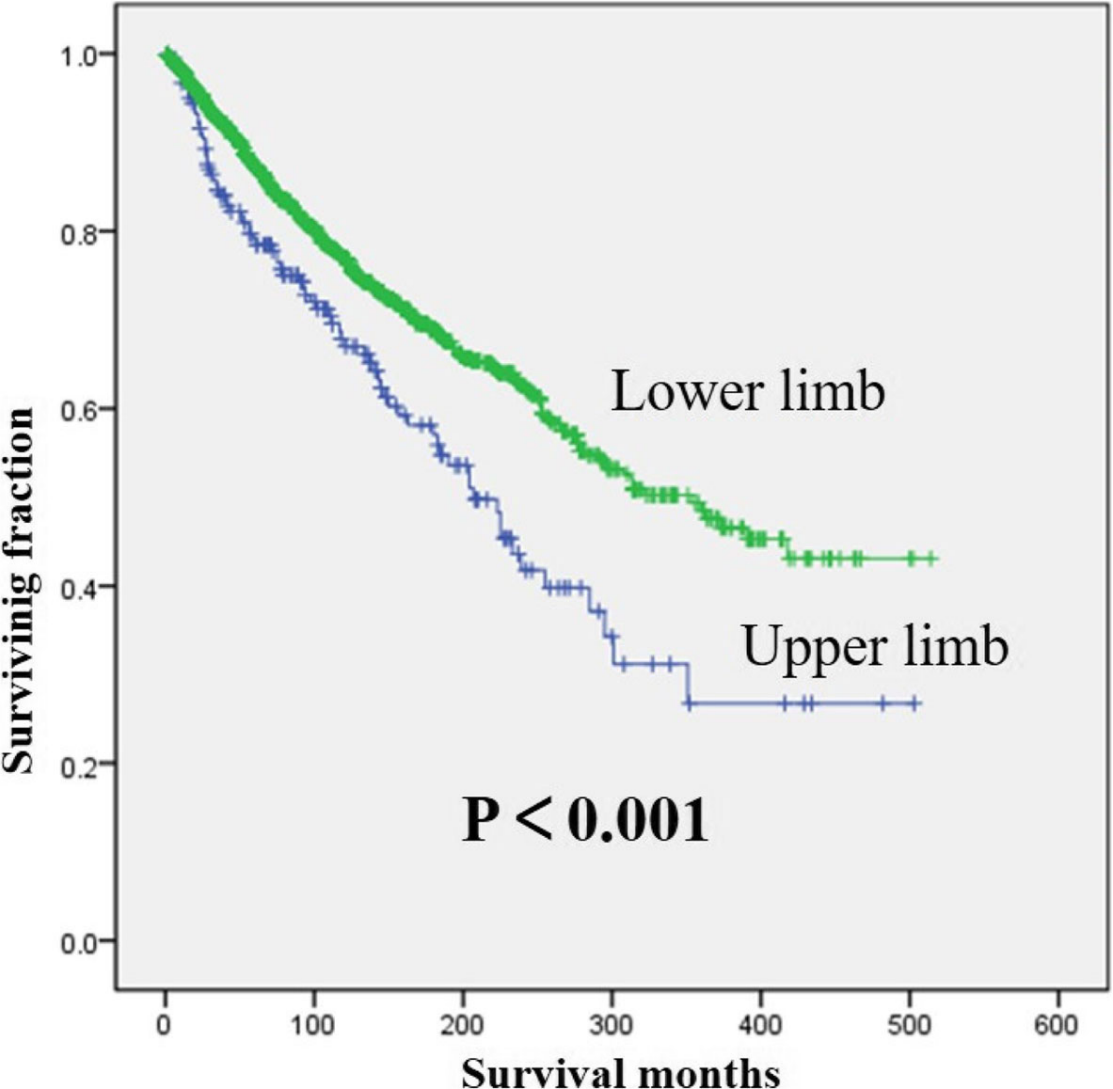
Kaplan–Meier method estimated OS in patients with extremity myxoid liposarcomas stratified by tumor site

**Fig. 3:**
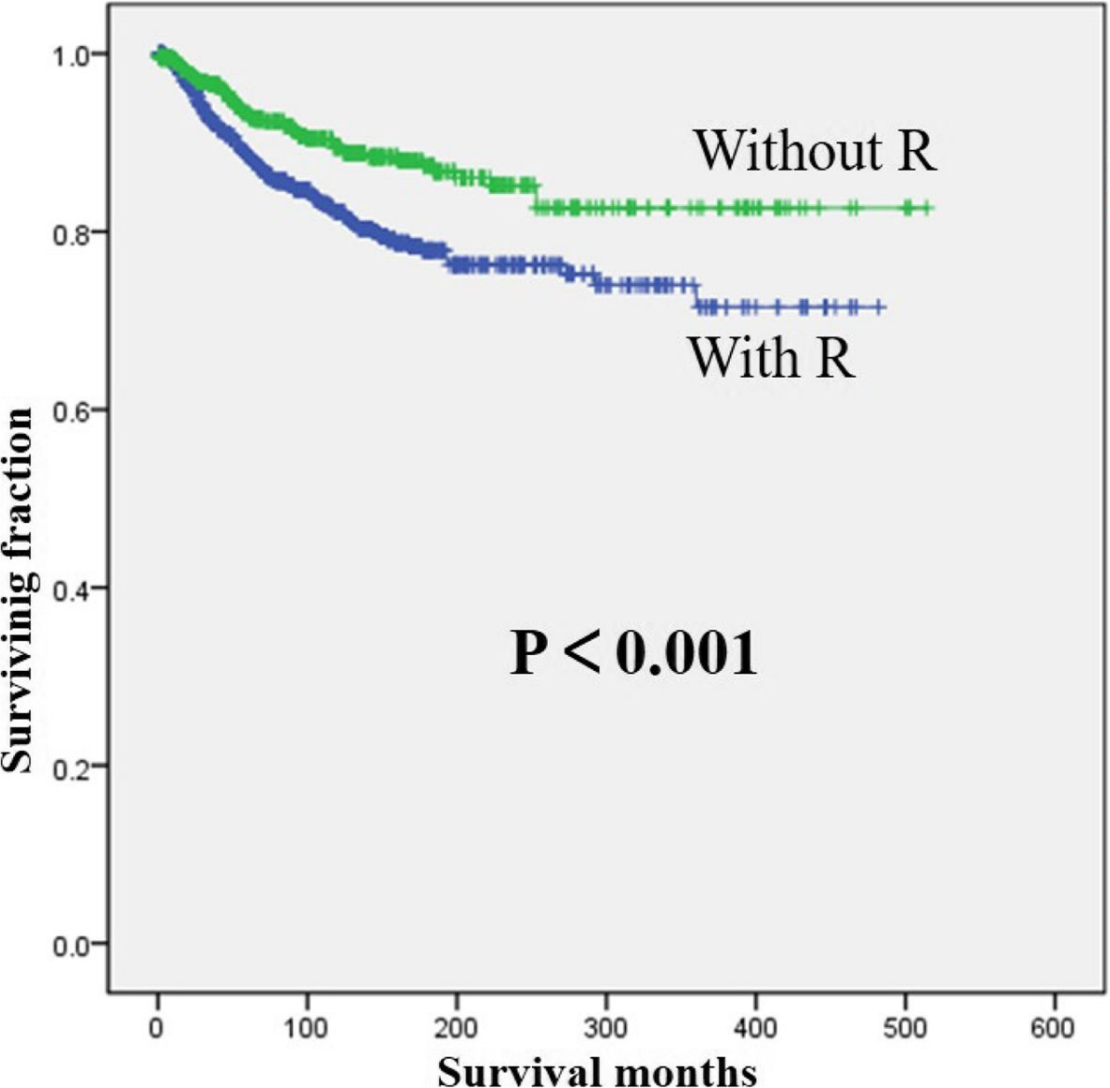
Kaplan–Meier method estimated CSS in patients with extremity myxoid liposarcomas stratified by radiation treatment. (R, radiotherapy)

Univariate analyses are shown in Table 2. This study revealed that the year of diagnosis and radiation treatment were not associated with OS. Age, gender, tumor grade, and tumor size were associated with significant differences in both OS and CSS. Upper limb site has been significantly associated with a decreased OS (Fig. 2). But CSS showed no significant difference based on tumor site. Patients who received radiation treatment had significantly worse CSS than those who did not (Fig. 3).

**Table 2:** Univariate analysis of variables in patients with myxoid liposarcoma of extremities after surgery using Kaplan–Meier method

Category	OS (log-rank *p* value)	CSS (log-rank *p* value)
Age at diagnosis	< 0.001	< 0.001
> 60 years vs 30–60 years	< 0.001	< 0.001
> 60 years vs < 30 years	< 0.001	< 0.001
30–60 years vs < 30 years	< 0.001	< 0.001
Gender	0.003	0.002
Year of diagnosis (< 2000 vs ≥ 2000)	0.766	0.018
Location (upper limb vs lower limb)	< 0.001	0.286
Tumor grade (low vs high)	< 0.001	< 0.001
Tumor size	< 0.001	< 0.001
> 10 cm vs 5–10 cm	0.001	< 0.001
> 10 cm vs < 5 cm	< 0.001	< 0.001
5–10 cm vs < 5 cm	0.718	0.136
Radiation treatment (yes vs no)	0.477	< 0.001

*OS* overall survival, *CSS* cancer-specific survival

### Multivariate analyses of independent predictors of OS or CSS among patients with extremity MLS

Multivariate analyses were employed to identify independent risk factors for survival in patients with extremity MLS (Table 3). On multivariate analysis of all patients, older age, male, high tumor grade, and tumor size > 10 cm were found to be independent risk factors of decreased OS and CSS. Additionally, year of diagnosis ≥ year 2000 was significantly associated with an increased CSS. Radiation treatment failed to become an independent risk factor for either OS or CSS.

**Table 3:** Multivariate analysis for OS and CSS for patients with myxoid liposarcoma of extremities after surgery

Variable	OS		CSS	
	Hazard ratio (95% CI)	*p* value	Hazard ratio (95% CI)	*p* value
Age (years)				
< 30	1		1	
30–60	6.435 (3.174–13.047)	< 0.001	5.690 (2.319–13.959)	< 0.001
> 60	21.512 (10.573–43.770)	< 0.001	14.699 (5.855–36.902)	< 0.001
Gender				
Female	1		1	
Male	1.329 (1.097–1.610)	0.006	1.497 (1.104–2.029)	0.009
Year of diagnosis				
< 2000	1		1	
≥ 2000	1.023 (0.816–1.284)	0.842	0.519 (0.376–0.716)	< 0.001
Location				
Upper limb	1		1	
Lower limb	0.820 (0.633–1.062)	0.132	0.820 (0.517–1.301)	0.400
Tumor grade				
Low	1		1	
High	2.281 (1.771–2.937)	< 0.001	3.259 (2.282–4.655)	< 0.001
Tumor size				
< 5 cm	1		1	
5–10 cm	0.992 (0.697–1.414)	0.967	1.489 (0.717–3.093)	0.286
> 10 cm	1.651 (1.166–2.338)	0.005	4.036 (2.008–8.111)	< 0.001
Radiation treatment				
Yes	1		1	
No	1.031 (0.847–1.255)	0.761	0.792 (0.572–1.095)	0.158

*OS* overall survival, *CSS* cancer-specific survival

## Discussion

Liposarcoma represents one of the most common sarcomas found in adults, characterized by adipocyte differentiation [12, 13]. Previous studies have focused on the recurrence and metastasis of extremity LPS and rarely performed survival analysis.

In addition, LPS subtype is one of the most important prognostic factors affecting survival, and extremity LPS is not a single entity [9]. Therefore, different LPS subtypes should be analyzed separately for their prognostic patterns and characteristics. This study is the largest research to describe the clinical features of extremity MLS and explore possible predictors of survival using the SEER program database. Among patients with extremity MLS, age ≥ 30 years, male gender, high tumor grade, year of diagnosis ≥ year 2000, and tumor size > 10 cm were independently associated with worse survival. Although radiotherapy achieved local control of tumor, it seemed to have no benefit for prolonging survival among patients with extremity MLS.

Previous studies found few MLS patients developed distant metastasis, and the tumor was generally low grade [14]. MLS patients experienced a survival advantage compared to dedifferentiated and pleomorphic LPS patients [4, 15, 16]. Nishida et al. retrospectively reviewed 53 patients with extremity and trunk MLS and reported the 5- and 10-year disease-specific and disease-free survival rates were 90% and 83%, and 77% and 77%, respectively [14]. We also observed encouraging estimated 5- and 10-year OS and CSS of 86.4% and 75.9%, 90.5% and 85.2%, respectively. However, Salduz et al. reported that the disease-free survival at 5 and 10 years were both 66%, whereas OS at 5 and 10 years were 78.1% and 71.0%, respectively [2]. The small sample size (23 patients) of their study and 5 cases with round cell components in the excision specimens may explain this difference.

MLS are usually diagnosed in adults and rarely in children. This study showed that 85.6% with extremity MLS were aged over 30. A previous report demonstrated age > 60 years independently predicted worse overall and disease-free survival of pure extremity and trunk MLS [14]. This study further revealed that age > 30 years was an independent risk factor of decreased OS and CSS, suggesting those patients need special consideration for treatment and follow-up. Some studies of LPS indicated that there were no differences in survival by gender [6, 14, 17]. However, this study identified male sex as an independent negative prognostic factor of both OS and CSS. Recently, Toulmonde et al. investigated retroperitoneal sarcomas and also found that male sex was a prognostic factor independently associated with poor OS [18]. Further studies will be needed to clarify the reasons for gender differences in survival.

Many studies analyzed LPS patients and identified that trunk tumor location was associated with a poorer outcome compared with an extremity location [19, 20]. However, the difference in prognosis between upper and lower limbs was not analyzed. In this study, although univariate analysis showed that lower limb was significantly associated with a decreased OS, it was not an independent prognostic factor of OS. Tumor grade was generally recognized as a very important predictor of prognosis. Muratori et al. reported that tumor grade was a significant risk factor affecting OS of MLS [13]. Similarly, this study revealed that tumor grade was an independent prognostic factor of both OS and CSS. Previous studies reported that the larger tumor size was associated with poorer prognosis and decreased survival rate of LPS patients. Salduz et al. found that tumor size > 15 cm was significantly associated with increased overall mortality [2]. Similarly, this study revealed that tumor size > 10 cm was an independent prognostic factor of both OS and CSS. However, Nishida et al. and Oh et al. reported that tumor size was not associated with survival [14, 19]. Maybe patients with larger tumor size had more local recurrence and metastasis and resulted in poor prognosis. Additionally, this study found that year of diagnosis ≥ year 2000 predicted better CSS on multivariate analysis, suggesting that treatment for extremity MLS has been gradually improved.

Surgery, radiotherapy, and chemotherapy constitute the current treatments of LPS patients. However, the appropriate treatment for extremity MLS patients remains controversial. Chemotherapy-related toxicity was as considerable and generally higher for older patients. Therefore, the treatment for older extremity MLS patients remains a challenge. As MLS is a radiosensitive tumor, radiotherapy can offer effective local control and reduce the local recurrence [5, 21]. Combination of surgery and perioperative RT is now widely adopted as the mainstream treatment for extremity STS [22].

In this study, more than half of the patients received radiotherapy. However, the effects of radiotherapy on survival of MLS patients especially extremity MLS patients are rarely studied. Kachare et al. reported that RT was associated with improved survival for high-grade sarcoma of the extremity [23]. However, Nishida et al. found that RT had no impact on either overall or disease-free survival of MLS [14]. Although our univariate analysis revealed that patients who received radiation treatment had significantly worse CSS than those who did not, it was not an independent prognostic factor of CSS. Radiotherapy is usually used in worse cases of high tumor grade and large tumor size. So, we performed stratification analysis and found that the effect of “no benefit of radiation therapy on survival” also presented in the different tumor grades or sizes. Different diagnostics and therapy forms might be used in different decades, which may affect prognosis. Due to diagnostic techniques were limited in earlier years, the tumor grading might be higher and the possibilities of radiation therapy might be weaker at that time. Since the number of cases before year 2000 was relatively small, we divided the year of diagnosis into before year 2000 and after year 2000. However, stratification analysis showed that the effect of “no benefit of radiation therapy on survival” also presented before or after year 2000. Additionally, some studies reported that patients with extremity STSs could achieve local control with surgical treatment without RT, suggesting RT should be a highly selective practice for extremity STS [7, 14]. Thus, RT seemed to have no benefit for prolonging survival of patients with extremity MLS. However, with the development of radiotherapy technology, further studies are urgently needed to confirm the effect of RT on the outcome and survival of extremity MLS.

### Limitations

There are several limitations of this investigation. First, this study was a retrospective study from a large secondary database, which does not provide access to detailed clinical information. Prospective study should be performed to further confirm our conclusion. Second, the SEER database does not include other important information such as time to recurrence during follow-up, radiotherapy regimen, and molecular pathological characteristics, which may affect the prognosis of patients. These variables may be an effective complement to this study, which will be an important section of our future research. Besides, a high percentage of unknown data about tumor grade (30.5%) was shown in this study which is regarded as an important prognostic factor. Although this study is helpful for doctors to make decisions, it did not include all prognostic factors and cannot always provide precise prognosis in clinical practice. Despite these limitations, our large sample size along with demographic and tumor data allows for the investigation of important associations and predictors of extremity MLS. Additionally, the SEER database provides high statistical power due to the collection of data from multiple centers.

## Conclusion

This is the largest population-based study to describe the demographics and analyze the prognosis for 1756 patients with extremity MLS. We identified age, gender, tumor grade, year of diagnosis, and tumor size as independent prognostic factors for OS and CSS in patients with extremity MLS. This study may help clinicians to better understand the features and prognosis of extremity MLS and to provide appropriate treatment recommendations.

## Abbreviations

CI: Confidence intervals
CSS: Cancer-specific survival
HR: Hazard ratios
ICD-O-3: International Classification of Diseases for Oncology, 3rd edition
LPS: Liposarcoma
MLS: Myxoid liposarcoma
OS: Overall survival
RT: Radiotherapy
SEER: Surveillance, Epidemiology, and End Results
STS: Soft tissue sarcoma
WHO: World Health Organization
